# Does the Surgical Approach Influence Bleeding in Hip Fracture Patients Taking Clopidogrel?

**DOI:** 10.7759/cureus.48559

**Published:** 2023-11-09

**Authors:** Enver Kılıç, Olgun Bingöl, Guzelali Ozdemir, Baran Sarıkaya, Atahan Durgal, Taha E Karahan

**Affiliations:** 1 Orthopaedics and Traumatology, Ankara Bilkent City Hospital, Ankara, TUR

**Keywords:** transfusion, bleeding, approach, clopidogrel, hemiarthroplasty, hip fracture

## Abstract

Background

The aim of this study was to evaluate the effect of the choice of approach on bleeding in patients with femoral neck fractures who are on clopidogrel.

Materials and methods

The study included patients over the age of 60 who were taking clopidogrel and had hemiarthroplasty surgery for a femoral neck fracture. A total number of 61 patients were evaluated in the study. Patients who underwent surgery using the posterior approach were assigned to group 1, while those who underwent surgery using the anterolateral approach were assigned to group 2. Preoperative and postoperative hemoglobin levels, transfusion needs, red blood cell (RBC) loss, duration of surgery, and the length of hospitalization were evaluated.

Results

The mean age of the patients was 79.36 ± 7.72 years. Twenty-nine patients were included in group 1 and 32 patients were included in group 2. There was no significant difference between the two groups in terms of gender, age, and comorbidities (p=0.74, p=0.12, p=0.23, respectively). There were no significant differences between group 1 and group 2 in terms of duration of surgery and length of hospital stay (p=0.41, p=0.37, respectively). Also, there was no significant difference in RBC loss between group 1 and group 2 (p=0.37).

Conclusion

The use of anterolateral or posterior approaches has no effect on bleeding in clopidogrel-treated patients having hemiarthroplasty for femoral neck fracture. The authors recommend surgeons choose the approach according to their experience and patients' needs.

## Introduction

The incidence of hip fractures in elderly patients is increasing every year. It is estimated that up to 6 million hip fractures will occur in 2050 [1]. Clopidogrel is an antithrombotic drug that works by inhibiting platelet function and is frequently used for cardiovascular, cerebrovascular, and peripheral vascular disorders. Studies have shown a high incidence of cardiovascular disease in patients with hip fractures. About 5% of patients with hip fractures use clopidogrel [2,3]. Patients receiving antithrombotic therapy such as clopidogrel have a higher risk of bleeding and complications [4].

Clopidogrel is the inhibitor of P2Y12 receptor, which is a target for adenosine diphosphate binding and platelet activation. The half-life of clopidogrel is eight hours and it inactivates platelets for a lifespan of 5-7 days. The feature that distinguishes clopidogrel from other antithrombotic is that there is no method for reversing its effect and the effect of fresh platelet transfusion is controversial [5].

It is known that patients with hip fractures using clopidogrel have a high risk of perioperative bleeding and spinal hematoma in case of regional anesthesia. There are many studies in the literature on the timing of surgery and early surgery is recommended [6]. It is also known that the surgical approach may affect the amount of perioperative blood loss and transfusion needed. To our knowledge, there is no study on the effect of surgical approaches on bleeding in this patient group. The aim of this study was to evaluate the effect of the posterior and anterolateral approach on bleeding in patients with femoral neck fractures on clopidogrel.

## Materials and methods

In this study, hip fracture patients admitted to Ankara Bilkent City Hospital between January 2017 and January 2022 were retrospectively evaluated. After approval of the Institutional Ethics Committee (Ankara Bilkent City Hospital Ethics Committee E1-20-1103), patient databases were reviewed. All of the researchers who participated in the study signed the most recent version of the Helsinki Declaration. Informed consent forms were obtained from all of the patients in the study.

Patients over the age of 60 who underwent hemiarthroplasty for femoral neck fracture between January 2017 and January 2022 and were taking clopidogrel were included in the study. Patients with multiple traumas, pathological hip fractures, neuromuscular diseases, hematological diseases, cognitive dysfunction, and other antithrombotic use were excluded. Also, patients who underwent total hip replacement were excluded from the study.

Between January 2017 and January 2022, a total of 1291 patients were admitted to our hospital with femoral neck fractures. Sixty-four of these patients were using clopidogrel and treated with hemiarthroplasty. Three patients whose data could not be accessed from the records were excluded from the study. A total of 61 patients were evaluated in the study. Among these patients, those who were operated through the posterior approach were included in group 1 and those who were operated through the anterolateral approach were included in group 2.

Clopidogrel was stopped during hospitalization in all patients and hemiarthroplasty surgeries were performed 72 hours after clopidogrel was stopped. The choice of approach had no clear criteria and was based on the surgeon's preference in each case.

All data were collected from the hospital record system. Age, gender, surgical approach, preoperative, intraoperative and postoperative transfusion, preoperative, postoperative, postoperative day 1,2,3 and discharge hemoglobin levels, red blood cell (RBC) loss, ASA (American Society of Anesthesiology) scores, duration of surgery, comorbidities and length of hospital stay were evaluated. Erythrocyte loss was evaluated by the Mercuriali formula.

Statistical analysis

Statistical analysis was performed by using SPSS 22.0 version software program for Windows (IBM Corp., Armonk, USA). Shapiro-Wilks test was used to determine parameters distribution. Kruskal-Wallis test was used for the comparison of qualitative data between the groups and Mann-Whitney U test was used to determine the difference between the groups. The results were evaluated within the 95% confidence interval and p<0.05 was considered statistically significant.

## Results

The study involved 61 patients, including 22 (36%) males and 39 (64%) females. The mean age of the patients was 79.36 ± 7.72 years. Twenty-nine patients (47.5%) (10 (34.5%) males, 19 (65.5%) females) were operated through the posterior approach and included in group 1, and 32 patients (52.5%)(12 (37.5%) males, 20 (62.5%) females) were operated through the anterolateral approach and included in group 2. The comorbidities of the patients are listed in Table 1. There was no significant difference between the two groups in terms of gender, age, and comorbidities (p=0.74, p=0.12, p=0.23, respectively). Almost all of the patients' ASA scores were 2 or higher. The mean ASA scores of groups 1 and 2 were 2.91 ± 0.87 and 2.79 ± 0.78, respectively. There was no difference between the ASA scores of the groups (p=0.29). 

**Table 1:** Comorbidities of the patients TIA: Transient Ischemic Attack

Comorbidities	Group 1 (n=29)	Group 2 (n=32)
Cardiovascular disease	25	28
Stroke/TIA	16	18
Diabetes	11	10
Renal disease	2	3
Malignancy	2	1
Smoking	3	2
Other	4	6

The mean preoperative, postoperative, and postoperative days 1, 2, 3, and discharge hemoglobin levels for groups 1 and group 2 are given in Table 2. The difference between preoperative and discharge hemoglobin levels was 1.3 ± 0.9 for group 1 and 0.9 ± 0.8 for group 2. Although the difference between preoperative and discharge hemoglobin levels was higher in group 1, this difference was not statistically significant (p=0.13).

**Table 2:** Comparison of groups in terms of hemoglobin levels, number of transfusions, length of stay in hospital, and duration of surgery Hgb: Hemoglobin *p<0.05: statically significant difference

	Group 1 (n=29)	Group 2 (n=32)	p-value*
Preoperative Hgb	10.8 ±1.9	11 ± 2.1	0.22
Postoperative Hgb	9.9 ± 1.3	10.4 ± 1.4	0.12
Postoperative day 1 Hgb	9.5 ± 1.1	10.2 ± 1.2	0.16
Postoperative day 2 Hgb	9.3 ± 1.1	9.9 ± 1.1	0.09
Postoperative day 3 Hgb	9.4 ± 1	10 ± 1.1	0.16
Discharge Hgb	9.5 ± 1.1	10.1 ± 1.2	0.12
Mean transfusion (unit)	1.1	0.9	0.39
Length of stay in hospital (days)	8.7 ± 4.4	7.9 ± 4.6	0.41
Operation time (minutes)	72 ± 20.3	67 ± 19.7	0.44

The mean duration of surgery of group 1 and group 2 was 72 ± 20.3 and 67 ± 19.7 minutes, respectively. There was no significant difference between group 1 and group 2 in terms of duration of surgery (p=0.41) (Table 2). The mean length of hospital stay for group 1 and group 2 was 8.7 ± 4.4 and 7.9 ± 4.6 days, respectively. There was also no significant difference in the length of hospital stay (p=0.44) (Table 2).

The mean total RBC loss was 1232 ml (1003-1586) for group 1 and 1066 ml (811-1417) for group 2. There was no significant difference in RBC loss between group 1 and group 2 (p=0.37) (Table 3). Furthermore, there was no significant difference in intraoperative and postoperative RBC loss between group 1 and group 2 (p=0.29, p=0.35, respectively).

**Table 3:** Comparison of groups in terms of RBC loss RBC: Red Blood Cell *p<0.05: statically significant difference

	Group 1	Group 2	p-value*
Total RBC loss (ml)	1232 (1003-1586)	1066 (811-1417)	0.37
Preoperative	0	0	
Intraoperative	881 (401-1293)	708 (369-1198)	0.29
Postoperative	351 (0-614)	358 (0-811)	0.35

## Discussion

This study showed that there was no difference in bleeding, transfusion rate, and length of hospital stay between hemiarthroplasty performed with posterior and anterolateral approaches in patients on clopidogrel with femoral neck fracture.

Clopidogrel is frequently used in the elderly population for cardiovascular and cerebrovascular diseases. In many studies, the use of clopidogrel in patients with hip fractures is 5-7% [2,3]. In this study, 4.9% of patients with femoral neck fractures were taking clopidogrel in accordance with the literature. And the mean number of comorbidities was 2. Almost all of the patients' ASA scores were 2 or higher.

Recovery of platelet function takes 5-7 days, which is the platelet life span. Elective surgeries can be safely performed 5-7 days after clopidogrel is stopped. No method to reverse the effect of clopidogrel has been described in the literature. However, in some cases, early surgery is recommended despite the risk of bleeding, and better results are obtained with early treatment of hip fractures [7]. Doleman and Moppett evaluated the patients on clopidogrel with femoral neck fractures. Early and delayed surgery were compared. They showed that there was no difference in mortality [8]. In many studies, patients on clopidogrel who underwent early, and delayed surgery were compared, there was no difference in mortality and the number of transfusions. However, the length of hospital stay increased for patients who underwent delayed surgery [9,10]. In other studies, it has been reported that in surgeries performed >7 days after discontinuation of clopidogrel, mortality increases at 30 days, 3 months, and one year after surgery [11-13]. In the current study, all patients were treated in the first 72 hours.

Mortality of the patients who underwent hemiarthroplasty due to femoral neck fracture is associated with length of hospital stay. Some studies report that the length of hospital stay of patients undergoing surgery increases after the first 48 hours [7,14]. Increasing length of hospital stay also increases costs. Shabat et al. showed that the hospital stay of the patients operated on in the first 48 hours was shorter and more cost-effective [15]. In our study, there was no difference in the hospital stay of the patients using clopidogrel who underwent hemiarthroplasty with posterior and anterolateral approaches.

Patients on clopidogrel and undergoing hemiarthroplasty for femoral neck fracture were included in the study. The risk of bleeding after hemiarthroplasty is higher compared to other implants. When patients undergoing intramedullary nailing and hemiarthroplasty were compared, it was shown that blood loss and transfusion requirement were higher in patients undergoing hemiarthroplasty [9]. In our study, mean blood loss was >1000 ml, and most of the loss was intraoperative. 

There are different approaches for hemiarthropathy, such as posterior and anterolateral. In the current study, we investigated whether there was a difference in bleeding and transfusion requirements in patients who used clopidogrel and underwent hemiarthroplasty with different approaches. There are studies comparing the blood loss of posterior and anterolateral approaches for patients who underwent hemiarthroplasty due to femoral neck fracture. Filippini et al. showed that in the posterior approach, there was less blood loss than in the anterolateral approach [16]. Tsailas et al. showed that there was no difference in blood loss between posterior and anterolateral approaches in patients who underwent hemiarthroplasty due to femoral neck fracture [17]. In the current study, there was no significant difference in bleeding between the anterolateral and posterior approaches. In most studies, the surgical time for the posterior approach is shorter than the anterolateral approach [18,19].

The current study has some limitations. First, our study has a retrospective design. Second, the number of patients included in the study was limited. Third, the effect of cemented and uncemented hemiarthroplasty on bleeding was not evaluated.

## Conclusions

The choice between anterolateral and posterior approaches does not impact bleeding in patients taking clopidogrel who undergo hemiarthroplasty for femoral neck fracture. Surgeons should select the approach based on their expertise and the specific requirements of the patients.

## References

[1] Cooper C, Campion G, Melton LJ 3rd (1992). Hip fractures in the elderly: a world-wide projection. Osteoporos Int.

[2] Manaqibwala MI, Butler KA, Sagebien CA (2014). Complications of hip fracture surgery on patients receiving clopidogrel therapy. Arch Orthop Trauma Surg.

[3] Johansen A, White J, Turk A (2008). Clopidogrel therapy--implications for hip fracture surgery. Injury.

[4] Iavecchia L, Safiya A, Salat D (2016). Impact of implementing a protocol on the perioperative management in patients treated with antithrombotics admitted for hip fracture surgery: an observational study. Basic Clin Pharmacol Toxicol.

[5] Li C, Hirsh J, Xie C, Johnston MA, Eikelboom JW (2012). Reversal of the anti-platelet effects of aspirin and clopidogrel. J Thromb Haemost.

[6] Rosencher N, Bonnet MP, Sessler DI (2007). Selected new antithrombotic agents and neuraxial anaesthesia for major orthopaedic surgery: management strategies. Anaesthesia.

[7] Pailleret C, Ait Hamou Z, Rosencher N, Samama CM, Eyraud V, Chilot F, Baillard C (2017). A retrospective comparison between delayed and early hip fracture surgery in patients taking clopidogrel: same total bleeding but different timing of blood transfusion. Int Orthop.

[8] Doleman B, Moppett IK (2015). Is early hip fracture surgery safe for patients on clopidogrel? Systematic review, meta-analysis and meta-regression. Injury.

[9] Chechik O, Amar E, Khashan M, Kadar A, Rosenblatt Y, Maman E (2012). In support of early surgery for hip fractures sustained by elderly patients taking clopidogrel: a retrospective study. Drugs Aging.

[10] Cox G, Talbot C, Topp K, Templeton P (2009). Clopidogrel and proximal femoral fractures: does timing of surgery affect blood loss and length of admission? A preliminary study prior to multicenter trial. Eur J Trauma Emerg Surg.

[11] Zehir S, Zehir R, Sarak T (2015). Early surgery is feasible in patients with hip fractures who are on clopidogrel therapy. Acta Orthop Traumatol Turc.

[12] Harty JA, McKenna P, Moloney D, D'Souza L, Masterson E (2007). Anti-platelet agents and surgical delay in elderly patients with hip fractures. J Orthop Surg (Hong Kong).

[13] Maheshwari R, Acharya M, Monda M, Pandey R (2011). Factors influencing mortality in patients on antiplatelet agents presenting with proximal femoral fractures. J Orthop Surg (Hong Kong).

[14] Siegmeth AW, Gurusamy K, Parker MJ (2005). Delay to surgery prolongs hospital stay in patients with fractures of the proximal femur. J Bone Joint Surg Br.

[15] Shabat S, Heller E, Mann G, Gepstein R, Fredman B, Nyska M (2003). Economic consequences of operative delay for hip fractures in a non-profit institution. Orthopedics.

[16] Filippini M, Bortoli M, Montanari A (2023). Does surgical approach influence complication rate of hip hemiarthroplasty for femoral neck fractures? A literature review and meta-analysis. Medicina (Kaunas).

[17] Tsailas PG, Argyrou C, Valavanis A (2021). Management of femoral neck fractures with the ALMIS approach in elderly patients: Outcomes compared to posterior approach. Injury.

[18] Martin R, Clayson PE, Troussel S, Fraser BP, Docquier PL (2011). Anterolateral minimally invasive total hip arthroplasty: a prospective randomized controlled study with a follow-up of 1 year. J Arthroplasty.

[19] D'Arrigo C, Speranza A, Monaco E, Carcangiu A, Ferretti A (2009). Learning curve in tissue sparing total hip replacement: comparison between different approaches. J Orthop Traumatol.

